# Human immunodeficiency virus-1 Tat exerts its neurotoxic effects by downregulating Sonic hedgehog signaling

**DOI:** 10.1007/s13365-022-01061-8

**Published:** 2022-02-18

**Authors:** Irfan A. khan, Arthur H. Worrad, Meera V. Singh, Sanjay B. Maggirwar, Vir B. Singh

**Affiliations:** 1 Department of Basic and Clinical Sciences, Albany College of Pharmacy and Health Sciences, Albany, NY 12208 USA; 2 School of Medicine and Dentistry, University of Rochester Medical Center, Rochester, NY 14642 USA; 3 Department of Microbiology, Immunology and Tropical Medicine, The George Washington University, Washington, DC 20052 USA

**Keywords:** HAND (HIV-associated neurocognitive disorder), Neuroinflammation, Blood–brain barrier, HIV-Tat, Sonic hedgehog

## Abstract

We previously showed that HIV-1 can alter the expression of tight junction proteins by downregulating Sonic hedgehog (Shh) signaling, thereby disrupting blood–brain barrier (BBB) integrity. In this study, we employed a conditional, CNS specific, Tat transgenic murine model to investigate if HIV-Tat exerts its neurotoxic effects by downregulating Shh signaling. Results indicate that Tat + mice exhibit significantly reduced expression of Shh and Gli1. HIV-Tat induced downregulation of Shh signaling correlated with disruption of BBB function and induced infiltration of peripheral leukocytes into the brain tissue. Further, our in vivo and in vitro experiments suggest that activation of Shh signaling can rescue detrimental effects of Tat on endothelial function by inducing the expression of junctional proteins and by decreasing the levels of inflammatory cytokines/chemokines.

## Introduction

Introduction of combined antiretroviral therapy (cART) has practically eradicated human immunodeficiency virus (HIV)-associated mortality and enabled HIV-infected individuals to live a near-normal life span. However, despite the use of cART, more than 50% of HIV-infected individuals are suffering with some form of HIV-associated neurocognitive disorder (HAND) (Becker et al. 2004; Heaton et al. 2011; Valcour et al. 2004). HAND is attributed to persistent neuroinflammation in CNS, driven by constant infiltration of HIV-infected/activated leukocytes from the periphery (Schleidgen et al. 2013). Various reports have recognized damage to blood–brain barrier (BBB) integrity as an early event following infection (Atluri et al. 2015; Eugenin et al. 2011; Strazza et al. 2011). BBB is comprised of specialized endothelial cells that are interconnected by tight junctions and supported by pericytes and astrocytic end feet that tightly regulate infiltration of peripheral factors including leukocytes into the CNS (Strazza et al. 2011).

In our earlier studies, we have shown that HIV infection can perturb BBB integrity by downregulating Sonic hedgehog (Shh) signaling. Further, pharmacological replenishment of Shh signaling by administration of smoothened agonist (SAG), a small molecule Shh mimetic, to chronically as well as acutely infected humanized mice resulted in rescue of BBB integrity and significantly reduced neuroinflammation, possibly by preventing the infiltration of infected/activated leukocytes into CNS (Singh et al. 2016, 2017). In adult brain, Shh is mainly secreted from astrocytes as a cleaved soluble factor. Further, Shh engages with Patched (PTCH) receptor present on endothelial cells that causes release of smoothened (SMO) which further activates Gli-1 transcription factor to induce the expression of tight junction proteins. Smoothened agonist (SAG) can directly bind to SMO and cause the activation of downstream signaling to reinforce BBB integrity by inducing the expression of tight junction proteins (Aruni et al. 2012; Osterlund and Kogerman 2006).

HIV-induced disruption of BBB may be mediated by HIV-encoded regulatory protein trans-activator of transcription (Tat) which has been shown to decrease the expression of tight junction proteins in brain microvascular endothelial cells. In a study from our group, we demonstrated that administration of recombinant Tat can destabilize BBB integrity in mice within 18 h post-exposure (Bruno et al. 2012). HIV-1 Tat possesses neurotoxic properties and mediates BBB dysregulation as well as activation of CNS resident cells including astrocytes and microglia. Even though HIV-infected individuals on cART have undetectable levels of virus, low level of Tat production persists throughout the infection and is suggested to play a central role in chronic inflammation, including neuroinflammation and leading to progressive neurodegeneration with accelerated aging (Allavena et al. 2018; Dickens et al. 2017). Thus, there is need to understand the toxic effects of HIV-1 Tat specifically on BBB and CNS resident cells and identify potential therapeutic targets to alleviate HIV-1 Tat-mediated neurotoxicity.

In this study, we hypothesize that HIV-1-induced BBB disruption as well as neuroinflammation can be attributed to HIV-1 Tat’s ability to disrupt Shh signaling in CNS. We utilized conditional, Tat-expressing (CNS specific) transgenic murine model to find that consistent expression of Tat in CNS can cause BBB disruption by dysregulating Shh signaling. HIV-1 Tat-induced BBB disruption was significantly rescued by inducing Shh signaling. Further, HIV-1 Tat-induced adverse effects in brain endothelial cells were rescued via boosting Shh signaling by SAG or recombinant Shh (rShh) treatments. Thus, this study suggests that manipulating Shh signaling can serve as a useful therapeutic approach to alleviate Tat-specific adverse effects in the cART era.

## Materials and methods

Experiments on HIV-1 Tat transgenic mice were carried out in accordance with the Animal Welfare Act and National Institute of Health (NIH) guidelines, and the University Committee on Animal Resources of the University of Rochester Medical Center approved the animal protocol (protocol# 2005–161).

### Mouse experiments

HIV-1 Tat + and Tat − mice (3 weeks old) were used. Tat transgenic mice were originally generated by Hauser and colleagues (Hauser et al. 2009). Tat + Transgenic mice conditionally express HIV-1 Tat protein (1–86 amino acids), driven by GFAP promoter; thus, Tat is expressed in a CNS targeted manner. GFAP Tet − on promoter is induced by consumption of doxycycline in the chow (Harlan laboratories, catalog # TD.01306). Tat − mice did not express HIV-1 Tat; instead they only expressed a doxycycline responsive rtTA transcription factor. Both the groups of mice (3 weeks old) were subjected to doxycycline diet for a period of 3 weeks and were used to perform various analyses. Further, Tat^+^ mice were also administered with 100 µl smoothened agonist (SAG; Cayman Chemicals, catalog# 11,914) intraperitoneally (20 µg/g body weight) on day 15 and day 18 after initiating the doxycycline diet.

#### Brain-infiltrating leukocyte (BIL) assay

BILs were isolated and quantitated as a marker for BBB integrity in Tat^+^ vs Tat^−^ mice. Isolation and quantitation of BILs were performed as described previously (Moller et al. 2012). Purified BILs were resuspended in PBS and fixed as described (Singh et al. 2017). The cells were stained with antibodies against CD45, a pan-leukocyte marker. The numbers of CD45^+^ cells in brain were measured via volumetric analysis (Accuri C6 Cytometer, Ann Arbor, MI).

#### Cell culture and in vitro treatments

Human brain microvascular endothelial cells (HBMECs; Cell System (catalog# ACBRI 376) were cultured in DMEM medium with 10% FBS, 1% penicillin/streptomycin, and 1 μg/ml hydrocortisone. Recombinant Tat − was provided by Dr. Steve Dewhurst at University of Rochester Medical Center. Cultured HBMECs were treated with purified HIV-1 Tat protein (500 nM) and SAG (500 nM), alone or in combination for various time points to analyze MAP kinases (30 min), mRNA transcripts (6 h), and protein levels (24 h).

Protein was extracted by lysing the cells in RIPA lysis buffer (catalog# 89,900; Thermo Fisher) supplemented with a mixture of protease and phosphatase inhibitors and incubated on ice for 15 min. RNA extraction was performed using TRIZOL reagent (catalog# 15,596–026; Invitrogen) as per manufacturer’s protocol.

#### Preparation of brain lysate

Brain lysates were prepared by homogenizing brain tissues using a motorized homogenizer (Cole-Parmer) in lysis buffer (20 µl/g of tissue) containing 50 mM HEPES (pH 7), 250 mM NaCl, 0.1% Nonidet P-40, 5 mM EDTA, 10 mM NaF, 0.1 mM Na_3_VO_4_, 50 μM ZnCl_2_, supplemented with 0.1 mM PMSF, 1 mM DTT, and a mixture of protease (Thermo Scientific, catalog# 78,430) and phosphatase inhibitors (Thermo Scientific, catalog# 78,420). Further, brain homogenates were incubated at 4 °C for 2 h on an orbital shaker. Lysates were centrifuged at 13,000 rpm for 20 min at 4 °C. The supernatants were carefully aspirated and transferred to newly labeled tubes and were further used in western blot assays.

#### Western blot analysis

Cell and brain tissue lysates were separated on 7.5–12% polyacrylamide gels under denaturing conditions. Proteins were transferred onto nitrocellulose membrane (Thermo Scientific, catalog #88,018) and blocked for 1 h in blocking buffer (LI-COR, catalog #927–40,000). Subsequently membrane was incubated with primary antibodies raised against Sonic hedgehog (rabbit monoclonal, Santa Cruz, catalog #2207), Gli-1 (rabbit polyclonal, Santa Cruz, catalog# sc-20687), smoothened (mouse monoclonal, Santa Cruz, catalog #sc166685), Occludin (goat polyclonal, Santa Cruz, catalog # sc-8035), claudin-5 (rabbit polyclonal, Santa Cruz, catalog # sc28670), ICAM-1 (rabbit polyclonal, Santa Cruz, catalog #sc7891), and Zo-2 (rabbit polyclonal, Santa Cruz, catalog #sc11448).

### RT-qPCR

Total RNA was extracted from mouse brain tissues and HBMECs using TRIzol Reagent (Invitrogen, catalog # 15,596,018); 1 μg RNA was digested with RNase-free DNase (Invitrogen, catalog# 18,068,015) and used as template to synthesize cDNA using the iScript cDNA synthesis kit (BIO-RAD). Expression levels of *IL-1β*, *CCL-2*, and *IL-6* were determined using real-time quantitative RT-PCR in a 10 μl reaction using TaqMan assays. BIO-RAD CFX 96 thermocycler was used for all the real-time quantitative PCRs. Expression levels were analyzed using the ΔΔCt method.

### Statistical analyses

GraphPad Prism version 7 was used to perform all statistical analyses. All datasets were tested for normal distribution of data using Shapiro–Wilk normality test. If the datasets passed normality test, then they were analyzed using unpaired *t* test or ordinary one-way ANOVA followed by Tukey’s multiple comparisons test. If the data did not pass normality test, then they were analyzed by Mann–Whitney *U* test or Friedman test followed by Dunn’s multiple comparisons test. All comparisons were done using unpaired *t*-test to compare group means or group variance, respectively. Statistical significance is indicated in the figures as *p < 0.05 and **p < 0.01.

## Results

### HIV-1 Tat disrupts BBB integrity by downregulating Shh signaling

**Fig. 1:**
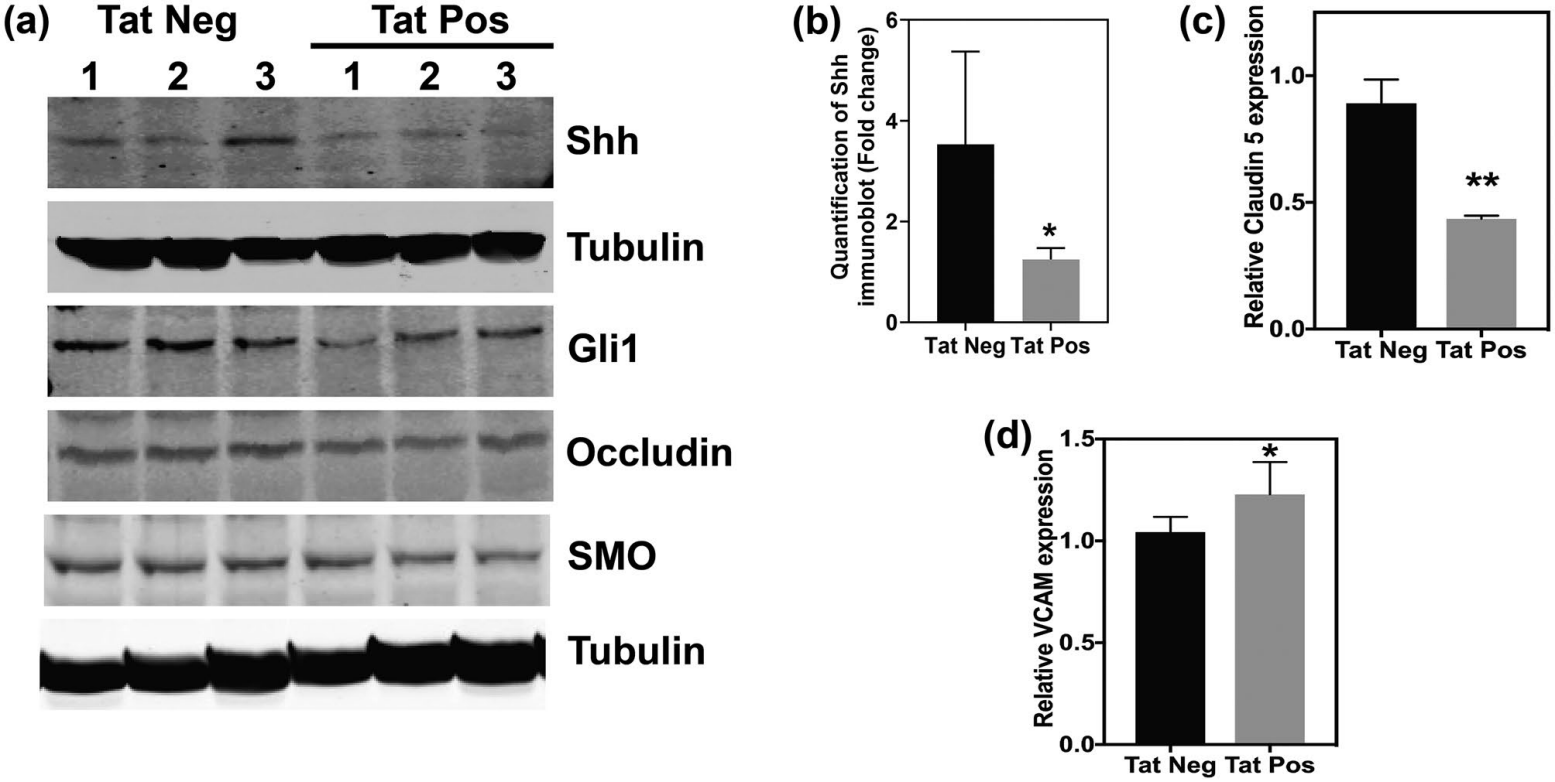
Brain-specific expression of HIV-Tat downregulates Shh signaling and BBB integrity determinants. **a** Immunoblots showing the expression of Shh, Gli-1, Occludin, and Smoothened in the brain lysates of Tat-positive and Tat-negative transgenic mice (*n* = 3 per group). Tubulin was used as internal control. **b** Quantification of Shh levels shown immunoblot in Fig. 1a relative to tubulin. **c** Expression of claudin-5 (*n* = 3 per group) and **d** Vcam1 was detected by performing RT-qPCR on RNA isolated from brains of Tat-positive and Tat-negative mice (*n* = 5 per group). Double asterisk indicates *p* < 0.01 and single asterisk indicates *p* < 0.05 using unpaired *t* test

**Fig. 2:**
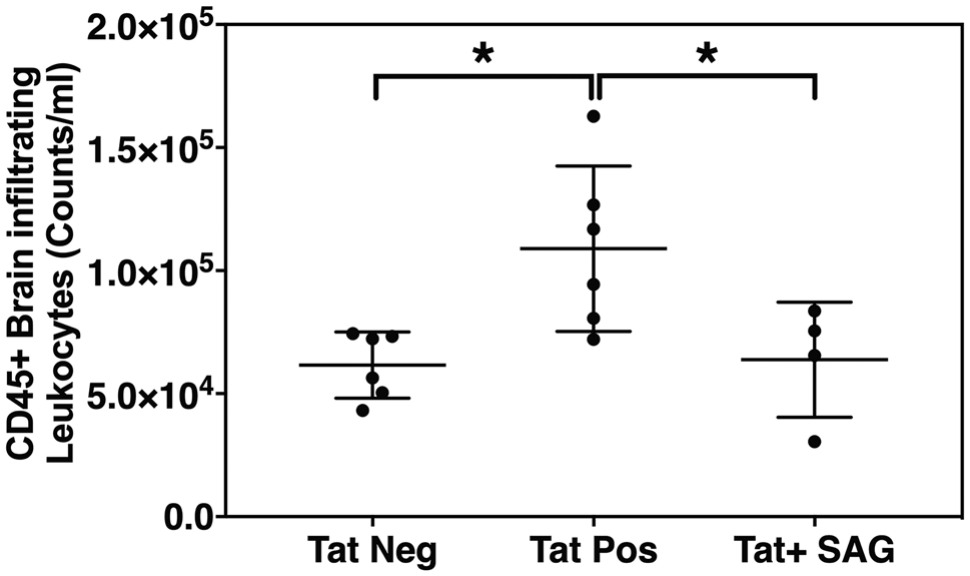
Re-enforcement of Shh signaling diminishes the HIV-Tat-induced infiltration of peripheral leukocytes into CNS. Brain tissues were isolated from all the three experimental groups and were homogenized to make single cell suspension, and subsequently detection of brain-infiltrating leukocytes was carried out by flow cytometry using antibody against CD45, a pan leukocyte marker. Single asterisk indicates *p* < 0.05 using ordinary one-way ANOVA followed by Tukey’s multiple comparisons test (*n* = 4–6 per group)

HIV-1 Tat expression was induced by supplementing mice with doxycycline containing food for 21 days. Subsequently, expression of Shh and Gli-1 was analyzed in mouse brain lysates. HIV Tat + mice demonstrated significantly lower levels of Shh as well as Gli-1 (Fig. 1a, b; *n* = 3 per group) suggesting Tat-mediated downregulation of Shh signaling. We did not observe any significant changes in the expression of smoothened (Fig. 1a). Further, downregulation of Shh signaling resulted in reduction of tight junction protein (claudin-5) and increase in the expression of adhesion molecule (Vcam-1) that are hallmarks of BBB dysregulation (Fig. 1c, *n* = 3 per group; Fig. 1d, *n* = 5 per group). Compromise in BBB integrity was measured in terms of increased levels of brain-infiltrating leukocytes in Tat + mice as compared to control Tat − mice (Fig. 2; *n* = 4–6 per group). Interestingly, rescue of Shh signaling by administration of SAG (2 doses; intraperitoneally at day 15 and day 18 after initiating doxycycline diet) significantly reduced the number of brain-infiltrating leukocytes (Fig. 2). These results suggest that Tat disrupts Shh signaling thereby altering BBB integrity which can be rescued by pharmacological boosting of Shh signaling.

### SAG can rescue the adverse effects of HIV-1 Tat in human brain microvascular endothelial cells

**Fig. 3:**
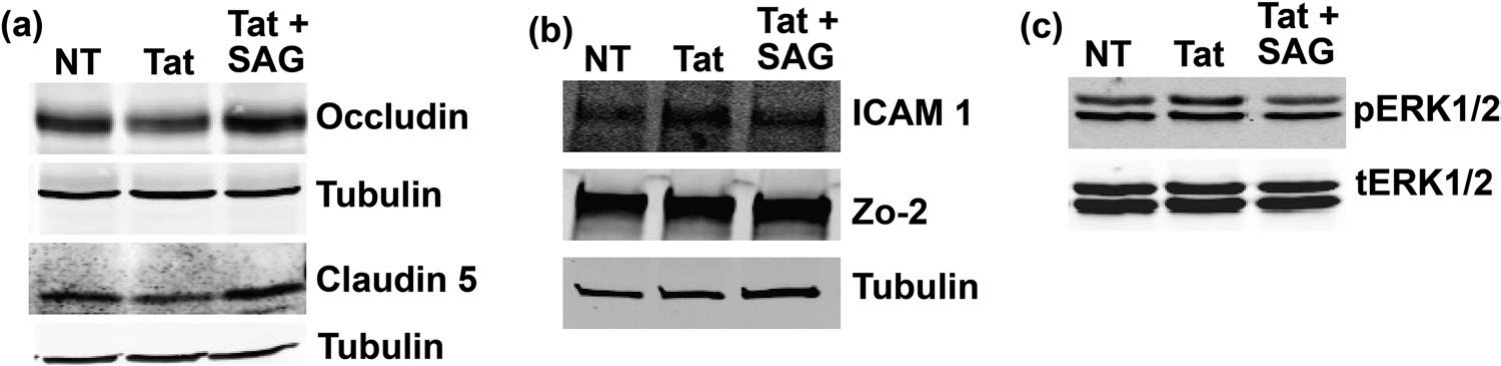
SAG can rescue the adverse effects of HIV-tat in HBMECs. **a** and **b** Representative immunoblots (*n* = 3) showing expression of tight junction proteins (claudin-5, Occludin, and Zo-2) and adhesion molecule (Icam-1) in HBMECs either non-treated or subjected to Tat treatment (500 nM) alone or in the presence of Smoothened agonist (500 nM). **c** phosphorylation status of ERK1/2 was detected by immunoblotting using phosphorylation-specific antibody (*n* = 3)

Human microvascular endothelial cells were treated with recombinant HIV-1 Tat (500 nM) for 24 h, and expression of tight junction proteins (claudin-5, Occludin, and Zo-2) as well as adhesion molecule ICAM-1 was detected by immunoblotting (*n* = 3). Results showed that HIV-1 Tat-induced downregulation of claudin-5 and Occludin (Fig. 3a) was rescued by SAG; on contrary HIV-1 Tat-induced upregulation of ICAM-1 was significantly reduced by SAG, whereas Zo-2 levels remained unchanged (Fig. 3b). Thus, these results suggest that SAG can counteract HIV-1 Tat and rescue brain endothelial cell function by strengthening tight junctions and reducing the expression of adhesion molecules.

### SAG rescues HIV-1 Tat-induced ERK1/2 activation

Previous reports have suggested that HIV-1 Tat can downregulate the expression of tight junction proteins via inducing MAP kinase activity specifically ERK1/2 (Andras et al. 2005). We measured the phosphorylation levels of ERK1/2 following HIV-1 Tat exposure for 15 min. Results showed that HIV-1 Tat-induced activation of ERK1/2 was significantly inhibited by co-treatment with SAG (Fig. 3c; *n* = 3).

### HIV-1 Tat-induced expression of pro-inflammatory mediators can be rescued by smoothened agonist

**Fig. 4:**
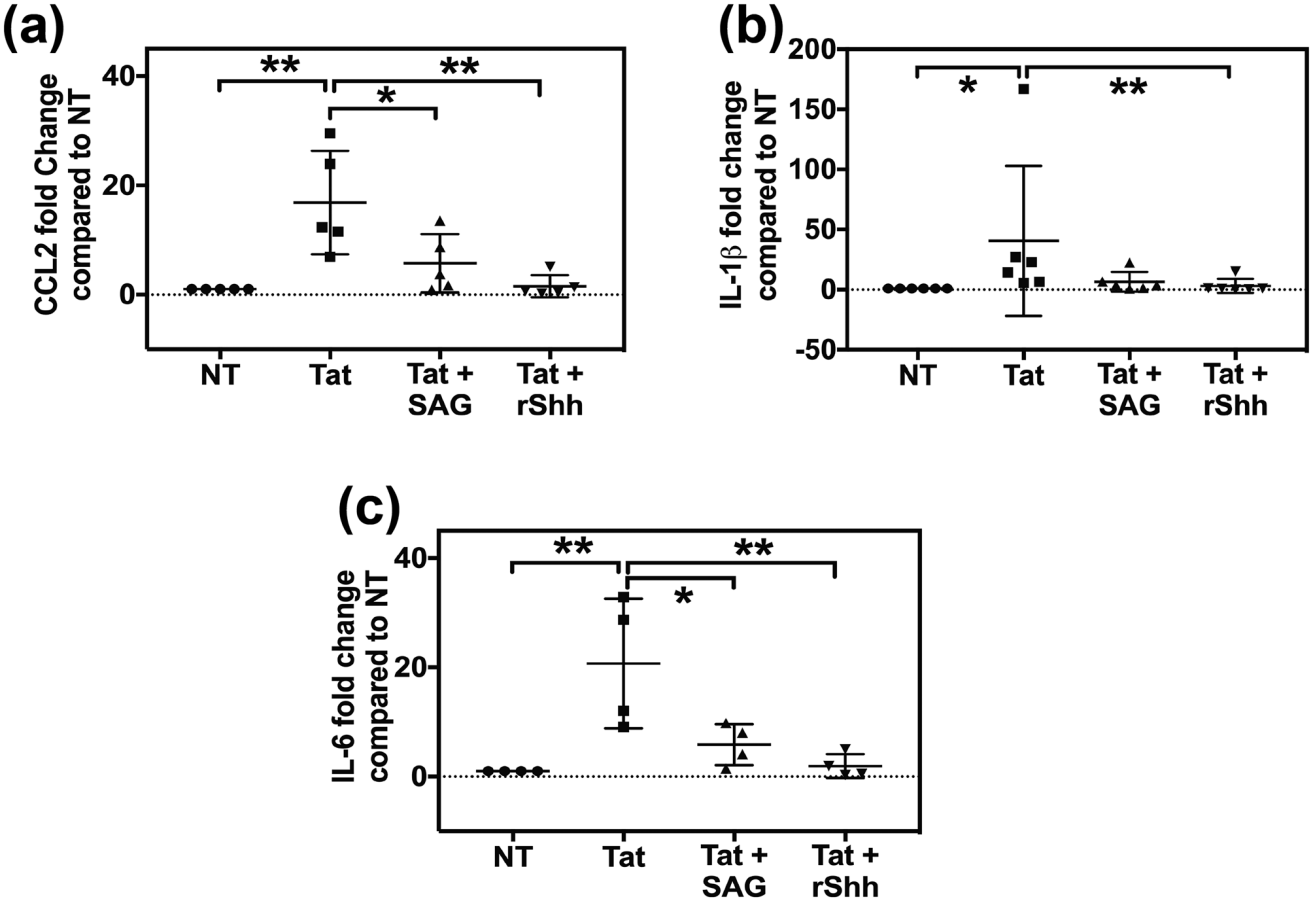
HIV-Tat-induced cytokine expression can be diminished by modulating Shh signaling. HBMECs were treated with HIV-Tat (500 nM) alone or in combination with SAG (500 nM) or rShh for 6 h (*n* = 4–6). Further total cellular RNA was collected and used to perform RT-qPCR using TaqMan assays to measure the expression of **a** CCL2, **b** IL-1β, and **c** IL-6. Double asterisk indicates *p* < 0.01, and single asterisk indicates *p* < 0.05 using Friedman test followed by Dunn’s multiple comparisons test

HIV-1 Tat has been reported to drive neuropathogenesis via inducing the infiltration of leukocytes through the BBB by downregulating the tight junction proteins as well as inducing the expression of inflammatory cytokines and chemokines. Thus, we analyzed the effect of Shh agonists on HIV-1 Tat-induced activation of inflammatory molecules, namely CCL2 (also known as MCP-1, monocyte chemoattractant protein-1), IL-6 (Interleukin-6), and IL-1β. Human brain endothelial cells were treated with HIV-1 Tat alone or in combination with SAG or rShh for 6 h. Gene expression was performed by RT-qPCR assays using TaqMan probes for respective genes. Results indicated pronounced activation of cytokines following the Tat treatment was significantly rescued by SAG or rShh treatments (Fig. 4; *n* = 4–6). Overall, these results suggest that modulation of Shh signaling may alleviate HIV-1 Tat-induced neuroinflammation by reinforcing the BBB tight junctions as well as diminishing the release of inflammatory cytokines, thus limiting the leukocyte infiltration and progression of neuroinflammation.

## Discussion

To the best of our knowledge, this is the first report which shows that HIV-1 protein Tat mediates its neurotoxic effects via downmodulation of Shh signaling. Tat is known to cause (i) BBB disruption (Andras and Toborek 2011; Banks et al. 2005; Xu et al. 2012), (ii) induction of inflammatory cytokines and chemokines (Bethel-Brown et al. 2012; Dhillon et al. 2007), (iii) astrocytosis (Zhou et al. 2004), and (iv) microglial activation (Minghetti et al. 2004) that subsequently contribute to the vicious cycle of neuroinflammation fueled by transmigration of infected/activated leukocytes into CNS from periphery (Park et al. 2001). Recently, we have demonstrated that HIV-1 infection in humanized mice can disrupt BBB integrity by specifically downregulating Shh signaling. In this report, we further identified HIV-1 Tat as the viral component that can mediate the downregulation of Shh signaling resulting in BBB dysfunction (Singh et al. 2016, 2017), using a transgenic mouse model that expresses HIV-1 Tat specifically in CNS compartment. Evidently, rescue of Shh signaling by SAG can alleviate HIV-1 Tat-induced BBB damage and diminish the transmigration of leukocytes into CNS.

Sonic hedgehog signaling is essential for normal brain development (Ingham and McMahon 2001), but its role in adult brain has not been very well studied. Astrocytes serve as the main source of Shh in adult brain. Astrocytic Shh engages Patched receptor present on endothelial cells allowing the activation of smoothened that causes induction of Gli-1 transcriptional activity which, in turn, leads to expression of tight junction proteins and maintenance of BBB integrity (Allahyari et al. 2019; Alvarez et al. 2011). Induction of Shh signaling has been reported to provide neuroprotection by strengthening BBB, reducing astrogliosis, and improving neuronal survival and repair (Alvarez et al. 2011; Chechneva et al. 2014; Iwata et al. 2005; Shao et al. 2017). Concomitantly, Shh has been reported to be neuroprotective in animal models of neurodegenerative disorders including Parkinson’s disease, ischemic stroke, and multiple sclerosis (Alvarez et al. 2011; Chechneva et al. 2014; Shao et al. 2017). A recent report has suggested that forebrain stab injury in rat model can specifically downregulate the Shh expression in astrocytes proximal to the injury site in a spatiotemporal manner. On similar lines, ours was the first group to demonstrate that HIV infection can downmodulate Shh signaling in humanized mice. Further, pharmacologic induction of Shh signaling by SAG in HIV-infected humanized mice provided neuroprotection against HIV-induced BBB disruption (Singh et al. 2016, 2017). As an extension of these previous studies, in this report, we demonstrate that HIV-1 Tat is the virologic factor responsible for downregulation of Shh signaling in the CNS, and SAG treatment can rescue BBB function in mice as well as in in vitro cultured HBECs. Our results show that expression of Tat in mouse brains resulted in decreased levels of Shh signaling proteins including Shh and Gli-1. This loss of Shh signaling can interrupt astrocyte-endothelial cross talk leading to decreased levels of tight junction proteins and induction of adhesion proteins in endothelial cells. Consequently, more leukocytes migrated from the periphery into the CNS resulting in neuroinflammation as seen by increased expression of CCL2, IL-1β, and IL-6 in Tat-treated ECs. All these detrimental effects of Tat could be reversed by activation of Shh signaling by SAG.

Lastly, HIV-1 Tat can downregulate the expression of tight junction proteins in brains endothelial cells via activation of MAP kinases including ERK1/2 (Andras et al. 2005). Consistently, we also observed induction of ERK1/2 phosphorylation by HIV-1 Tat exposure. Interestingly, HIV-1 Tat-induced ERK1/2 phosphorylation was significantly diminished by co-treatment with SAG. This observation is very intriguing, and we are currently working on investigating the cross-talk between Shh signaling and ERK1/2 signaling cascade. So far, only a couple of reports have suggested a potential cross-talk between Shh signaling and MAPKs via non-canonical pathways leading to transcriptional activation of Gli transcription factors (Barakat et al. 2013; Lauth and Toftgard 2007). Ongoing studies will provide information on the possible interaction between Shh signaling mediators and ERK1/2 and its impact on BBB and CNS homeostasis. Altogether, our study provides evidence to suggest that HIV-1 Tat might be exerting neurotoxic effects via dysregulating Shh signaling and pharmacological modulation of this signaling may provide beneficial outcomes to control incidences and severity of HAND in HIV-infected population.
